# Convalescent plasma therapy for COVID-19 patients: a protocol of a prospective meta-analysis of randomized controlled trials

**DOI:** 10.1186/s13063-021-05066-2

**Published:** 2021-02-01

**Authors:** Lajos Szakó, Nelli Farkas, Szabolcs Kiss, Szilárd Váncsa, Noémi Zádori, Nóra Vörhendi, Bálint Erőss, Péter Hegyi, Hussain Alizadeh

**Affiliations:** 1 Institute for Translational Medicine, Medical School, University of Pécs, Pécs, Hungary; 2 János Szentágothai Research Centre, University of Pécs, Pécs, Hungary; 3 Institute of Bioanalysis, Medical School, University of Pécs, Pécs, Hungary; 4 Doctoral School of Clinical Medicine, University of Szeged, Szeged, Hungary; 5 First Department of Medicine, Medical School, University of Szeged, Szeged, Hungary; 6 Division of Hematology, First Department of Medicine, Medical School, University of Pécs, Ifjúság street 13, Pécs, H-7624 Hungary

**Keywords:** COVID-19, SARS-COV-2, nCOV-2019, Convalescent plasma, Prospective meta-analysis

## Abstract

**Background:**

Coronavirus disease 2019 (COVID-19) is an infection with possible serious consequences. The plasma of recovered patients might serve as treatment, which we aim to assess in the form of a prospective meta-analysis focusing on mortality, multi-organ failure, duration of intensive care unit stay, and adverse events.

**Methods:**

A systematic search was conducted to find relevant registered randomized controlled trials in five trial registries.

A comprehensive search will be done continuously on a monthly basis in MEDLINE (via PubMed), Embase, Cochrane Central Register of Controlled Trials (CENTRAL), and Web of Science to find the results of previously registered randomized controlled trials. The selection will be done by two independent authors. Data extraction will be carried out by two other independent reviewers. Disagreements will be resolved by a third investigator.

An update of the search of the registries and the first search of the databases will be done on the 21st of July.

Data synthesis will be performed following the recommendations of the Cochrane Collaboration. In the case of dichotomous outcomes (mortality and organ failure), we will calculate pooled risk ratios with a 95% confidence interval (CI) from two-by-two tables (treatment Y/N, outcome Y/N). Data from models with multivariate adjustment (hazard ratios, odds ratio, risk ratio) will be preferred for the analysis. *P* less than 0.05 will be considered statistically significant. In the case of ICU stay, weighted mean difference with a 95% confidence interval will be calculated. Heterogeneity will be tested with *I*^2^, and *χ*^2^ tests. Meta-analysis will be performed if at least 3 studies report on the same outcome and population.

**Discussion:**

Convalescent plasma therapy is a considerable alternative in COVID-19, which we aim to investigate in a prospective meta-analysis.

## Background

There is currently an outbreak of respiratory disease caused by a novel coronavirus. The virus has been named “SARS-CoV-2,” and the disease it causes has been named “coronavirus disease 2019” (COVID-19). The outbreak has affected almost every country of the world, and as of 5 July 2020, a total of 11,046,917 confirmed cases and 526,465 deaths had been reported (www.who.int). Recently, two papers have been published reporting on efficient and safe vaccines, [1, 2] and several others are under investigation and approval in phase III. Since widespread vaccination takes time, alternative treatments are still needed in the early stage of the disaese. International randomized, controlled trials investigating the effect of treatments in patients hospitalized with COVID-19 have been launched (recovery and solidarity). The RECOVERY trial so far demonstrated the efficacy of dexamethasone in patients receiving either invasive mechanical ventilation or oxygen alone [3]. Among the treatment strategies under investigation is the administration of convalescent plasma collected from individuals who have recovered from COVID-19 [4–7]. Use of convalescent plasma was studied in outbreaks of other respiratory infections, including the 2003 SARS-CoV-1 epidemic, the 2009–2010 H1N1 influenza virus pandemic, and the 2012 MERS-CoV epidemic [8–10]. The effectiveness of convalescent plasma was highlighted in these studies, and none of the studies demonstrated adverse events. Therefore, it is essential to study the safety and efficacy of COVID19 convalescent plasma in clinical trials. Multiple published and unpublished studies have now reported on the use of convalescent plasma to treat severely or critically ill COVID-19 patients, without unexpected or severe adverse events. In the sole randomized controlled trial reported to date, critically ill but not intubated patients, receiving convalescent plasma showed more frequent and faster clinical improvement compared to controls. However, the trial was terminated early due to a lack of eligible patients at the study sites in China [11]. The results from this RCT, and many other systematic works, which were not conducted in a prospective manner support the concept that convalescent plasma should be used before COVID-19 is life-threatening to clear the virus more rapidly and avoid further tissue damage, rather than using this approach to treat patients with inflammatory end-stage organ failure [12–14]. Although none of these works aimed to include only randomized controlled trials in a prospective manner, but they provide quantitative synthesis of the available data. Multiple ongoing clinical trials are investigating the use of convalescent plasma in patients with less severe infection, or prophylactically in highly susceptible individuals, such as exposed health care workers or family caregivers of COVID-19 patients, situations predicted to result in more potential benefit from passive antibody transfer.

COVID-19 is a newly emerging disease, and there is not much evidence on its treatment. Thus, applying a prospective approach of a comprehensive evaluation of novel therapies is desirable, which can be achieved by the use of prospective meta-analysis (PMA). As the question requires sufficient statistical power, PMA also proves to be beneficial. Furthermore, as the hypotheses, selection criteria, and intended analyses are stated before the availability of the results of the actual randomized trials, it overcomes the limitations of traditional, retrospective meta-analyses [15].

We aim to assess the efficacy of convalescent plasma treatment of COVID-19 patients for the outcomes of mortality, multi-organ failure, and duration of intensive care unit (ICU) stay in a prospective meta-analysis.

## Methods

The protocol is based on the Preferred Reporting Items for Systematic review and Meta-Analysis Protocols (PRISMA-P) statement [16]. Throughout the review process, the recommendations of the Cochrane Handbook for Systematic Reviews of Interventions will be followed [17].

This protocol has been registered at PROSPERO International prospective register of systematic reviews in advance under the number of CRD42020197442.(https://www.crd.york.ac.uk/prospero/).

### Systematic search and selection of trial registries

**Fig. 1:**
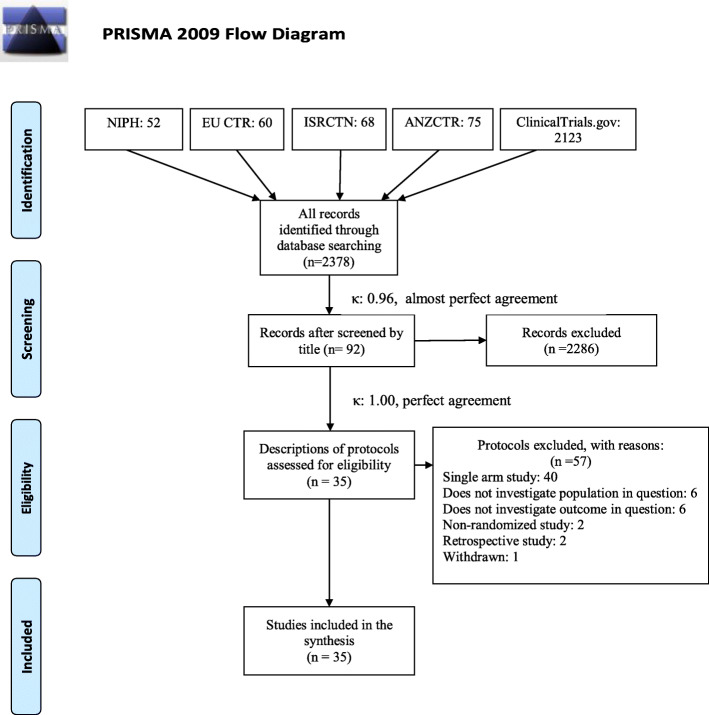
Prisma flowchart of the selection process and the results of Cohen’s kappa

A systematic search was carried out on 15 June 2020 with the following search key: COVID 19 OR “SARS-CoV2” OR “2019-nCoV” in the ClinicalTrials.gov, EU Clinical Trial Register, International Standard Randomised Controlled Trial Number (ISRCTN) registry, Australia and New Zealand Clinical Trial Registry (ANZCTR), and NIPH Clinical Trials Search registry to find eligible, registered randomized controlled trials. Two independent review authors performed the selection first based on the title, then based on the full protocol individually. In the case of disagreements, a third investigator was involved. A trial protocol proved to be eligible by title if it contained the term “plasma” in the context of intervention. A protocol was included in the level of full-text selection if it was a two-arm, randomized trial reporting on at least one of the populations and outcomes in question. All included patients should be PCR-confirmed COVID-19 cases, which will be divided into four subpopulations: P1: respiratory involvement (hypoxia, pneumonia, acute respiratory distress syndrome, requirement of oxygenation or ventilation); P2: patients admitted to the intensive care unit or are critically ill; P3: hospitalized patients without restriction on the severity, including mild, moderate, severe, and critically ill patients; P4: severe condition, defined as following the most recent World Health Organization (WHO) classification; intervention (I): convalescent plasma; control (C): placebo or any other active control; outcomes (O): mortality at any points of time after baseline, intensive care unit stay, multi-organ failure, adverse events. To quantify the level of agreement, Cohen’s kappa of both stages of the selection was calculated. The selection process and Cohen’s kappa results are presented in Fig. 1. Details of the included protocols are shown in Table 1. Reasons for exclusion on the level of full-text protocol are presented in Table 2.

**Table 1:** Basic characteristics of identified trial protocols

Registration number	Anticipated completion	Sample size	Plasma therapy details (dose, titer, etc.)	Comparison(s)	Outcomes		
					Mortality	MOF	ICU stay
**Adult COVID-19 patients with respiratory involvement (mechanical ventilation, pneumonia, ARDS, etc.)**							
NCT04405310	July 2020	80	250 mL of CP	Placebo	+	+	+
NCT04421404	April 2021	30	250 mL of CP	Non-immune control plasma	+	−	−
NCT04415086	May 2022	120	400 mL (300–600 mL) CP + SMT	200 mL (150–300 mL) CP + SMT	+	−	−
				SMT			
NCT04418518	December 2021	1200	500 mL of CP	No-CP	+	−	+
NCT04397757	November 2020	80	2 units of CP	SMT	+	+	−
NCT04374487	May 2021	100	200 mL of CP	SMT	+	+	−
NCT04428021	December 2021	180	3 units of CP on day 1,3,5 + SMT	3 units of standard plasma + SMT	+	+	+
				SMT			
NCT04356534	June 2020	40	400 mL of CP given as 200 mL over 2 h in 2 consecutive days + SMT	SMT	+	−	+
NCT04393727	October 2020	126	200 mL of CP	SMT	+	−	+
NCT04358783	May 2021	30	200 mL of CP	SMT	+	−	−
NCT04340050	November 2020	80	2 units of CP	SMT	+	+	−
NCT04385199	August 2020	30	200 mL of CP transfusion over 3 h	SMT	−	−	+
NCT04385186	December 2020	60	2 × 200 mL of CP	SMT	+	+	+
NCT04380935	August 2020	60	CP + SMT	SMT	+	+	+
**Adult COVID-19 patients admitted to the intensive care unit**							
NCT04391101	December 2021	231	2 units of CP (between 400 and 500 mL)	SMT	+	−	−
NCT04342182	July 2020	426	300 mL of CP	SMT	+	−	−
NCT04347681	April 2021	40	10–15 mL/kg CP daily, up to 5 days	No-plasma	+	−	+
NCT04381858	September 2020	500	400 mL (2 units) of CP	Human Ig	+	+	+
**Adult, severe COVID-19 patients**							
NCT04385043	May 2021	400	CP + SMT	SMT	+	−	−
NCT04403477	October 2020	20	400 mL of CP + SMT	200 mL of CP + SMT	+	+	+
				SMT			
NCT04364737	April 2023	300	250-500 mL of CP	Placebo	+	−	+
NCT04346446	May 2020	29	CP + SMT	Placebo + SMT	+	+	+
NCT04425915	May 2021	400	2 units of CP for 3 days + SMT	SMT	+	−	+
NCT04359810	April 2021	105	200–250 mL of CP	Standard plasma	+	−	−
**Adult, hospitalized COVID-19 patients**							
NCT04425837	February 2021	236	400 mL of CP + SMT	SMT	+	+	+
NCT04345991	June 2020	120	2 units of CP	SMT	+	−	−
NCT04388410	December 2020	250	2 units of CP	Placebo	+	−	−
NCT04366245	December 2021	72	CP	SMT	+	+	−
NCT04348656	December 2020	1200	500 mL of CP	SMT	+	−	+
NCT04345523	July 2020	278	CP	SMT	+	−	−
NCT04392414	September 2020	60	2 units of CP in 24 h	2 units of standard plasma in 24 h	+	−	+
NCT04383535	September 2020	333	10–15 mL/kg of CP (5–10 mL/h infusion rate)	Placebo + SMT	+	+	+
NCT04377568	May 2022	100	10 mL/kg (max 500 mL) of CP + SMT	SMT	+	+	+
NCT04395170	June 2021	75	2 units of CP in 3 days	Anti-COVID-19 human Ig	+	−	+
				SMT			
NCT04344535	August 2021	500	450–550 mL of CP	450–550 mL of standard plasma	+	−	−

*MOF* multiorgan failure, *ICU* intensive care unit, *COVID-19* coronavirus disease 2019, *ARDS* acute respiratory distress syndrome, *CP* convalescent plasma, *SMT* standard medical therapy, *Ig* immunoglobulin

**Table 2:** Excluded protocols and the reason for exclusions

Protocol number	Reason for exclusion
NCT04354831	Non-randomized study
NCT04353206	Single-arm study
NCT04345679	Single-arm study
NCT04372368	Single-arm study
NCT04333355	Single-arm study
NCT04412486	Single-arm study
NCT04355897	Single-arm study
NCT04390178	Single-arm study
NCT04321421	Single-arm study
NCT04343755	Single-arm study
NCT04397523	Single-arm study
NCT04384497	Single-arm study
NCT04388527	Single-arm study
NCT04344015	Single-arm study
NCT04383548	Single-arm study
NCT04420988	Single-arm study
NCT04363034	Single-arm study
NCT04389710	Single-arm study
NCT04338360	Single-arm study
NCT04397900	Retrospective study
NCT04409184	Retrospective study
NCT04360278	Single-arm study
NCT04358211	Single-arm study
NCT04352751	Single-arm study
NCT04408209	Single-arm study
NCT04389944	Single-arm study
NCT04360486	Single-arm study
NCT04348877	Single-arm study
NCT04343261	Single-arm study
NCT04392232	Single-arm study
NCT04384588	Single-arm study
NCT04356482	Single-arm study
NCT04374565	Single-arm study
NCT04384588	Single-arm study
NCT04374565	Single-arm study
NCT04377672	Single-arm study
NCT04332380	Single-arm study
NCT04357106	Single-arm study
NCT04354766	Non-randomized prospective cohort
NCT04327349	Single-arm study
NCT04407208	Single-arm study
NCT04361253	Children included
NCT04365439	Single-arm study
NCT04411602	Single-arm study
NCT04325672	Single-arm study
NCT04373460	Outpatient care
NCT04390503	Does not investigate population in question
NCT04375098	Does not investigate population in question
NCT04374526	Does not investigate population in question
NCT04323800	Does not investigate population in question
NCT04325672	Withdrawn
NCT04361253	Does not investigate the outcome in question
NCT04333251	Does not investigate the outcome in question
NCT04374370	Does not investigate the outcome in question
NCT04365439	Does not investigate the outcome in question
NCT04372979	Does not investigate the outcome in question
NCT04355767	Does not investigate the outcome in question

### Systematic search and selection of databases

A systematic search will be performed on 21 July 2020 in four scientific databases, MEDLINE (via PubMed), Embase, Cochrane Central Register of Controlled Trials (CENTRAL), and Web of Science, for randomized controlled trials (RCT). The following query will be used in all databases without any filters or restrictions: ((COVID 19 OR “SARS-CoV2” OR “2019-nCoV”) AND (plasma OR serotherapy OR “passive immun*”)). Reference lists of eligible articles and citing articles (via Google Scholar search engine) will also be screened to capture all relevant studies.

After the automatic and manual removal of duplicates using a reference management software (EndNote X9, Clarivate Analytics), two review authors will independently screen titles, abstracts, and full-texts against predefined eligibility criteria. A third review author will resolve any disagreements at each level of the selection process.

Inclusion criteria specified any RCTs that are reporting on the population and outcomes (mortality, multi-organ failure, duration of intensive care unit stay, adverse events) in question, as stated above. We will exclude non-randomized clinical trials and trials not reporting on the population and outcomes in question. In the case of overlapping study populations and updates, we will include the study with a higher patient number.

### Updates on the systematic searches

Regarding the trial registries and scientific databases, we intend to extract all records every month with the same methodology. The first systematic search and update on the trial registries will be on 21 July 2020.

### Data extraction

A standardized data extraction form will be developed a priori and will be piloted by the authors performing the data extraction. Two independent reviewers will extract data from all included studies. The following data will be extracted: first author, year of publication, study location, study design, study population, the type and details of interventions received, mean age, sex, number of patients in each group, inclusion criteria, and outcomes. Outcomes will include mortality, multi-organ failure, and intensive care unit stay. Discrepancies will be resolved by consensus and the involvement of a third author. All data will be compiled in an Excel spreadsheet (Office 365, Microsoft, Redmond, WA, USA) for analysis.

### Statistical analysis

Data synthesis will be performed using the methods recommended by the working group of the Cochrane Collaboration [11]. Data from models with multivariate adjustment (hazard ratios, odds ratio, risk ratio) will be preferred for the analysis. In the case of dichotomous outcomes (mortality and organ failure), we will calculate pooled risk ratios with a 95% confidence interval from two-by-two tables (treatment Y/N, outcome Y/N), if multivariate results are not available. *P* less than 0.05 will be considered statistically significant. Statistical analysis will be performed using random effects model. In the case of ICU stay, weighted mean difference with a 95% confidence interval will be calculated. Heterogeneity will be tested with *I*^2^, and *χ*^2^ tests, *p* less than 0.1, will indicate significant heterogeneity.

Meta-analysis will be performed using STATA v.16 (StataCorp. 2019, College Station, TX: StataCorp LLC.), Comprehensive Meta-Analysis v.3 (Biostat 2013, Englewood NJ), and R v.4.0.0 (R Core Team 2020, Vienna, Austria) software if at least three studies of same outcomes, assessed at the same point of time after the baseline are available.

A Trial Sequential Analysis (TSA 0.9.5.10.) will also be performed to quantify the statistical reliability and to estimate the optimal information size (OIS), if it is possible. We plan to perform the TSA for every included outcome. In the case of the *Z* curve, a *p* value less than 0.05 will be considered significant.

The presence of publication bias will be assessed visually by examining a funnel plot, as well as statistically by using Egger’s regression method if at least 8 studies are available. We will also use the trim and fill method to address this question [18].

If possible, subgroups of treatment modalities (dose and administration of plasma), age (< 18 years, 18–65 year, > 65 years), gender, and comorbidities will be presented. In the case of missing data, the corresponding authors will be contacted, or if individual patient data is available the missing variables will be calculated.

### Risk of bias and certainty of the evidence

The quality of all the included studies will be independently assessed by two reviewers using the Revised Cochrane risk-of-bias tool for randomized trials (RoB 2) [19]. Bias will be evaluated in five distinct domains: randomization process, deviations from intended interventions, missing outcome data, measurement of the outcome, and selection of the reported results. Within each domain, one or more signaling questions will be answered, which will lead to the judgments of the level of risk of bias: low (low for all domains), some concerns (some concerns in at least two domains), and high (at least one domain or some concerns for multiple domains) risk of bias. The results of the risk of bias assessment will be summarized narratively with full assessments, furthermore, a figure describing the results will be also published. We plan to perform the risk of bias assessment for every included outcome. Any disagreements will be solved by discussion and the involvement of a third reviewer if necessary.

The quality of evidence will be assessed by the Grading of Recommendations Assessment, Development, and Evaluation (GRADE) system. The certainty of evidence will be classified into four levels: high, moderate, low, or very low. Evidence is downgraded by concerns about the risk of bias, imprecision, inconsistency, indirectness, or publication bias. Two independent reviewers will decide the overall quality of the evidence. A third review author will resolve disagreements.

### Patient and public involvement

No patients were or will be involved in the design, conduction, or interpretation of our review.

## Discussion

Convalescent plasma therapy might be a good alternative to prevent the negative effects of COVID-19, but the clear benefits remain unclear. A prospective meta-analysis from randomized controlled trials can fill this void in terms of mortality, need and duration of intensive care unit stay, and organ failure. Furthermore, this protocol might serve as a basis for the not widely used methodology of prospective meta-analysis. Although prospective meta-analyses might include individual patient data, we do not intend to do so, corresponding authors will be only contacted in the case of missing data.

## Data Availability

Data is available from the corresponding author upon reasonable request. We plan to publish our results in peer-reviewed journals.

## Abbreviations

COVID-19: Coronavirus disease 2019
SARS-COV-2: Severe acute respiratory syndrome coronavirus 2
nCOV-2019: Novel coronavirus 2019
CI: Confidence interval
Y/N: Yes/no
ICU: Intensive care unit
PRISMA-P: Preferred Reporting Items for Systematic review and Meta-Analysis Protocols
SARS-CoV-1: Severe acute respiratory syndrome coronavirus 1
MERS-CoV: Middle East respiratory syndrome coronavirus
RCT: Randomized controlled trial
PMA: Prospective meta-analysis
ISRCTN: International Standard Randomised Controlled Trial Number
WHO: World Health Organization
GRADE: Grading of Recommendations Assessment, Development, and Evaluation
